# H3K79me3T80ph is a Novel Histone Dual Modification and a Mitotic Indicator in Melanoma

**DOI:** 10.1155/2012/823534

**Published:** 2012-11-25

**Authors:** Danielle R. Martinez, Hunter W. Richards, Qiushi Lin, Carlos A. Torres-Cabala, Victor G. Prieto, Jonathan L. Curry, Estela E. Medrano

**Affiliations:** ^1^Huffington Center on Aging, Baylor College of Medicine, One Baylor Plaza, Houston, TX 77030, USA; ^2^Health Science Center, University of Texas, 6431 Fannin Street, Houston, TX 77030, USA; ^3^Department of Molecular & Cellular Biology, Baylor College of Medicine, One Baylor Plaza, Houston, TX 77030, USA; ^4^Department of Genome Dynamics, Lawrence Berkeley National Laboratory, University of California, 1 Cyclotron Road, Berkeley, CA 94720, USA; ^5^Department of Pathology, The University of Texas MD Anderson Cancer Center, 1515 Holcombe Boulevard, Houston, TX 77030, USA; ^6^Department of Dermatology, The University of Texas MD Anderson Cancer Center, 1515 Holcombe Boulevard, Houston, TX 77030, USA; ^7^Department of Dermatology, Baylor College of Medicine, One Baylor Plaza, Houston, TX 77030, USA

## Abstract

The current study characterizes the mitosis-associated histone dual modification on the core of histone H3: trimethylation of histone H3 lysine 79 and simultaneous phosphorylation of H3 threonine 80 (H3K79me3T80ph). Through the use of protein and microscopy-based techniques, we find that H3K79me3T80ph shares a similar spatial and temporal regulation as H3S10ph but additionally requires methyltransferase activity. In addition, we find that Aurora kinase activity is necessary for the catalysis of H3K79me3T80ph *in vivo*. Finally, our analysis of H3K79me3T80ph using a tissue microarray indicates that H3K79me3T80ph marks a subset of primary cutaneous melanomas with metastatic potential indicating that H3K79me3T80ph may identify a subset of invasive melanomas with a more aggressive clinical behaviour.

## 1. Introduction

The nucleosome is the fundamental unit of chromatin; it consists of an octamer of core histones proteins (2 molecules each of H3, H4, H2A, and H2B) around which DNA is wrapped. Core histones are characterized by an unstructured N-terminal tail and the histone fold motif. Lysine residues on histone H3 can be mono-, di-, or trimethylated (me1, me2, and me3 resp.), with the majority of H3 methylation marks residing on the N-terminal tail of H3 [1]. However, lysine methylation can also occur on an exposed region of the histone H3 core at lysine 79 (H3K79) [2]. H3K79 methylation is catalyzed by DOT1, which has been implicated in cell-cycle regulation in both yeast and mammals [2–5]. On the tail of H3, three methylatable lysines lie adjacent to residues that have been found to be phosphorylated during mitosis, T3/K4, K9/S10, and K27/S28 [6–8]. However, it is not clear in what capacity lysine methylation and the adjacent phosphorylations occur together during mitosis or what the role is of these methyl-phospho modifications. 

On the core of histone H3, methylatable K79 lies adjacent to threonine 80, which was found, in a mass-spectrometry screen, to be phosphorylated (H3T80ph) in an analysis of adult mouse brain [9]. To our knowledge no reports have directly reported H3T80 phosphorylation (with or without the neighbouring K79 methylation), making this the first examination of a histone dual modification consisting of H3K79 trimethylation and H3T80 phosphorylation (H3K79me3T80ph) in the chromatin environment. Using a monoclonal antibody putatively recognizing H3K79me3T80ph, we find that H3K79me3T80ph is primarily associated with mitotic chromosomes. However unlike H3S10ph, H3T80ph does not appear to be catalyzed by Aurora B. Finally, using a tissue microarray we find that H3K79me3T80ph labels a subset of primary cutaneous melanomas with metastatic potential.

## 2. Materials and Methods

### 2.1. Cell Culture

Culturing of UCD-Mel-N and IIB-Mel-N cells has been previously described [10]. HeLa cells were grown in DMEM supplemented with 10% fetal bovine serum. For cell synchronization experiments, cells were treated with 0.1 *μ*g/mL of colcemid (Roche) for 16 hours and with 10 *μ*M of MG132 for the last 3 hours of treatment. Mitotic cells were then isolated by mitotic shake off. ZM 447439 (Enzo Life Sciences) was used at 0.5, 1, and 2 *μ*M. When ZM447439 was used in conjunction with colcemid, treatments were added simultaneously and incubated for 16 hours.

### 2.2. Histone Purification

Histones were isolated by acid extraction as previously described [11]. 

### 2.3. Antibodies

Antitrimethyl (K79)-phospho (T80)-Histone H3, clone MC491 (Millipore) referred to as H3K79me3T80ph; anti-H3S10ph (Abcam); anti-Histone H3 (Abcam); anti-H3K79me3 (Abcam); anti-H3K79me2 (Abcam); anti-Tubulin (Abcam); anti-Cyclin B1 (Abcam); anti-Actin (Abcam).

### 2.4. Peptides

Biotinylated peptides having sequence homology to histone H3 amino acids 71–88 were generated, and the peptide sequence is as follows: Biotin-Val-Arg-Glu-Ile-Ala-Gln-Asp-Phe-Lys-Thr-Asp-Leu-Arg-Phe-Gln-Ser-Ser/Ala-Ala-OH. Unmodified, K79T80ph, K79me2T80, and K79me2T80ph peptides were generated by Genemed Synthesis. K79me1T80, K79me3T80, and K79me3T80ph were generated by Keck Biotechnology Resource Laboratory, Yale University. H3 (1–21) and biotinylated H3 (1–21) phospho-(S10) are commercially available (Anaspec).

### 2.5. Western Blotting

Western blotting was performed as previously described [12].

### 2.6. Immunofluorescence

Immunofluorescence was performed as previously described [12]. Fluorescent images were obtained using an Axio Imager M1 microscope and AxioCam MRm camera utilizing AxioVision Rel. 4.6.3 software (Carl Zeiss, Inc.).

### 2.7. Peptide Competition

25 *μ*g of acid-extracted histones were fractionated by SDS-PAGE and transferred to nitrocellulose membrane (Bio-Rad). 15 *μ*M of each peptide was preincubated with H3K79me3T80ph antibody for 1 hour. Membranes were then incubated with antibody/peptide mixture for 1 hour, and western blotting was completed using standard protocol. 

### 2.8. Peptide Dot Blot

Increasing amounts of peptides (50, 100, 150, 200, and 250 nmoles) were transferred to PVDF membrane (GE Healthcare Life Sciences) by the use of dot blot apparatus. H3K79me3T80ph western blotting was completed using standard method. To demonstrate equal peptide spotting, the membranes were incubated with Avidin-HRP, extensively washed, and detected using an ECL detection kit (Denville Scientific).

### 2.9. Flow Cytometry

1 × 10^6^ HeLa cells were isolated for each flow cytometry reaction. Cells were fixed in 1% formaldehyde and washed with PBS, and stored in 70% ethanol at −20°C for 24 hours. Fixed cells were permeabilized with 0.1% Triton-X-100 in PBS and blocked with 3% BSA in PBS. Primary antibody was incubated for 1 hour in 1% BSA, followed by a 30-minute incubation with Alexa Fluor-488 secondary antibodies (Invitrogen) in 1% BSA. For propidium iodide staining cells were incubated for 10 minutes with 0.1 mg/mL RNase A then stained with 10 *μ*g/mL of propidium iodide. All reactions were suspended in 1 mL of water prior to analysis.

Cells were analyzed at the Cytometry and Cell Sorting Core (Baylor College of Medicine), using a LSRFortessa (BD Biosciences). Data from 20,000 events were analyzed using FloJo software (Tree Star Inc.). Data bars represent the mean (± S.E.M), *n* = 3. Statistical analysis was performed using a Student's *t*-test; results were considered significant if *P* < 0.05.

### 2.10. *In Vitro* Kinase Assays

6 ng of Aurora B/INCENP (Millipore) was added to reaction buffer (20 mM Tris, pH 7.5, 1 mM EGTA, 10 mM MgCl, 1 mM NaF, 0.1 mM Na_3_VO_4_, 1 mM DTT, and 50 *μ*M ATP) with 30 *μ*M of peptide and 5 *μ*Ci of *γ*-^32^P-ATP (specific activity 2,500 Ci/mmol; Perkin Elmer) and was then incubated at 30°C for 30 min. Reactions were stopped by the addition of 3% phosphoric acid, and the products were captured on P81 nitrocellulose filters (Millipore). P81 filters were washed in 0.75% phosphoric acid and methanol and subsequently assayed for incorporation of ^32^P by scintillation counting.

### 2.11. Melanoma Tissue Samples and Array

After approval by the Institutional Review Boards for human subject research, formalin-fixed paraffin-embedded tissues were obtained from the archives of the Department of Pathology, Section of Dermatopathology at the University of Texas MD Anderson Cancer Center. 87 melanocytic lesions were obtained including benign nevi (10 lesions), primary cutaneous melanoma without metastasis (25 lesions) and primary cutaneous melanoma with metastasis (19 lesions), and metastatic melanoma (33 lesions). All primary melanomas analyzed in the tissue microarray (TMA) were obtained from areas separate from the original lesion and therefore were not local recurrences. All diagnoses were rendered by a dermatopathologist. Tissue array construction was performed as previously described [13].

### 2.12. Immunohistochemistry

Immunohistochemical studies were performed as previously described [12]. H3K79me3T80ph antibody was used at a 1 : 100 dilution.

### 2.13. Statistical Analysis of Melanoma Tissue Array

The number of anti-H3K79me3T80ph positive cells (mitotically active cells and cells with speckled nuclear pattern) per high-power field in the melanoma tissue array was scored by two dermatopathologists (J. L. Curry, C. Torres-Cabala). The intensity of labelling was scored as no labelling, weak, moderate, and strong. Associations between average number of H3K79me3T80ph positive cells and categorical variables were examined using the analysis of variance (JMP software, Cary). Results were considered significant if *P* < 0.05. Significance levels were not adjusted for multiple comparisons.

## 3. Results and Discussion

### 3.1. H3K79me3T80ph Antibody Specifically Recognizes Both Trimethylated Lysine and Phosphorylated Threonine

**Figure 1:**
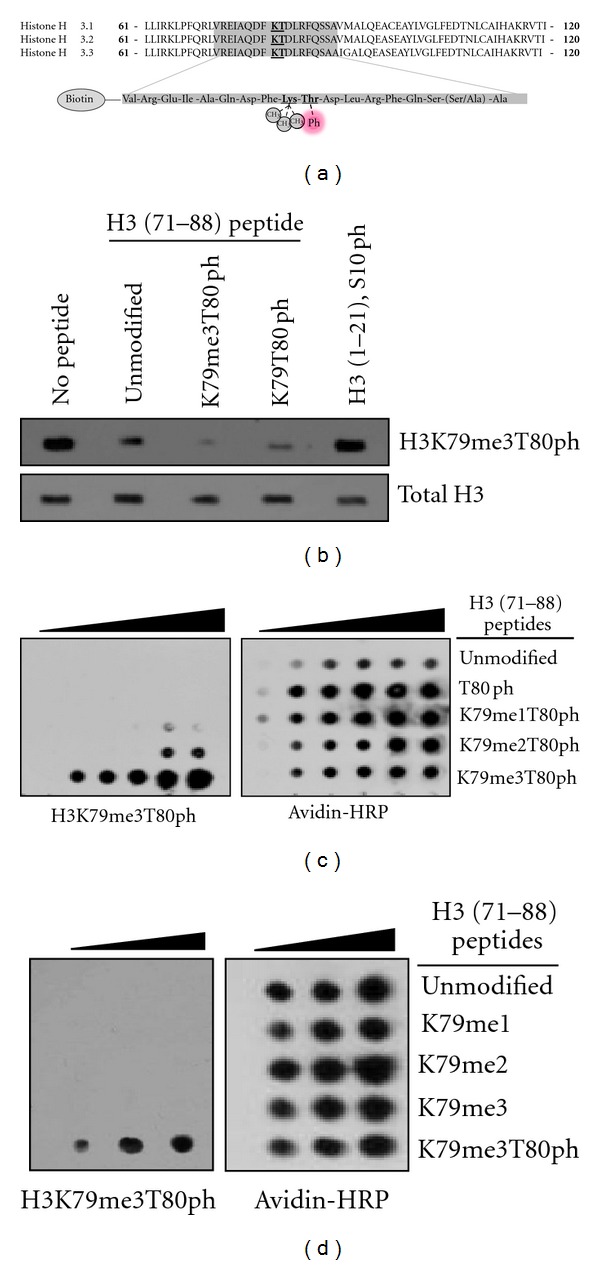
H3K79me3T80ph antibody specificity. (a) Shown are the three H3 isoforms and their amino acid sequence in the core of the protein surrounding lysine 79 and threonine 80 (emphasized) (top), and the biotinylated peptides designed for this study that are either unmodified, contain mono-, di-, or trimethylation of K79 plus phosphorylated T80, or phosphorylated T80 alone (bottom). (b) Histones from HeLa cells were isolated and subjected to western blotting with the H3K79me3T80ph antibody alone (no peptide) or in the presence of 15 *µ*M of biotinylated H3 (71–88) peptides with no modification, K79 trimethylation and T80 phosphorylation, T80 phosphorylation alone, or an H3 (1–21) S10ph peptide. (c) 50, 100, 150, 200, 250 nmoles of biotinylated H3 (71–88) peptides containing mono-, di-, or trimethylated K79 in conjunction with T80 phosphorylation were spotted onto nitrocellulose and subjected to H3K79me3T80ph and Avidin-HRP western blotting. (d) 50, 100, 150, 200, 250 nmoles of biotinylated H3 (71–88) peptides with K79 trimethylation and T80 phosphorylation or mono-, di-, or trimethylated K79 alone were spotted onto nitrocellulose and subjected to H3K79me3T80ph and avidin-HRP western blotting.

Although the dual modification H3K79meT80ph has been suggested, and the antibody for this modification is commercially available, to our knowledge no studies have been reported for this modification [14]. Therefore, we initiated experiments aimed at determining the specificity of this antibody. To do so, biotinylated peptides representing 18 amino acids of H3 (residues 71–88) and containing various levels of lysine methylation at lysine 79 (H3K79) in the presence and absence of phosphorylation at threonine 80 (H3T80) were generated (Figure 1(a)). A peptide competition assay was performed in which the H3K79me3T80ph antibody was either untreated (no peptide) or preincubated with H3 peptides containing modifications at K79me3T80ph, T80ph, or S10ph. The antibody/peptide mixture was then used to perform a western blot on extracted histones. Complete loss in signal was observed with the peptide containing both K79me3 and an adjacent T80ph showing that the antibody is highly specific for the dual H3K79me3T80ph modification (Figure 1(b)). An H3T80ph peptide successfully competed for antibody binding, indicating that the antibody may also recognize H3T80ph alone (Figure 1(b)). Having seen that the presence of H3T80ph alone can prevent antibody recognition of histones, we next examined the extent to which the H3K79me3T80ph antibody reacts with peptides containing different levels of K79 methylation adjacent to phosphorylated T80. Increasing molar amounts of peptides containing T80ph alone, and in combination with mono-, di-, or trimethylated K79 (K79me1T80ph, K79me2T80ph, and K79me3T80ph resp.), were spotted onto nitrocellulose and subjected to H3K79me3T80ph western blotting. The H3K79me3T80ph antibody had strongest immunoreactivity with the K79me3T80ph peptide (Figure 1(c)). In addition, the H3K79me3T80ph antibody reacts much more strongly with the K79me3T80ph peptide than the T80ph peptide, suggesting that the competition seen with the T80ph peptide in the peptide competition may be the result of decreased sensitivity in the immunoblot assay. To ensure that the H3K79me3T80ph antibody does not recognize lysine methylation in the absence of adjacent T80 phosphorylation, the dot blot analysis was repeated comparing a K79me3T80ph peptide to those containing methylated K79 alone (Figure 1(d)). Once again, the H3K79me3T80ph antibody shows a very specific immunoreactivity with the K79me3T80ph peptide and no reactivity against peptides containing only methylated K79.

### 3.2. H3K79me3T80ph Occurs Primarily in Proliferating Cells

**Figure 2:**
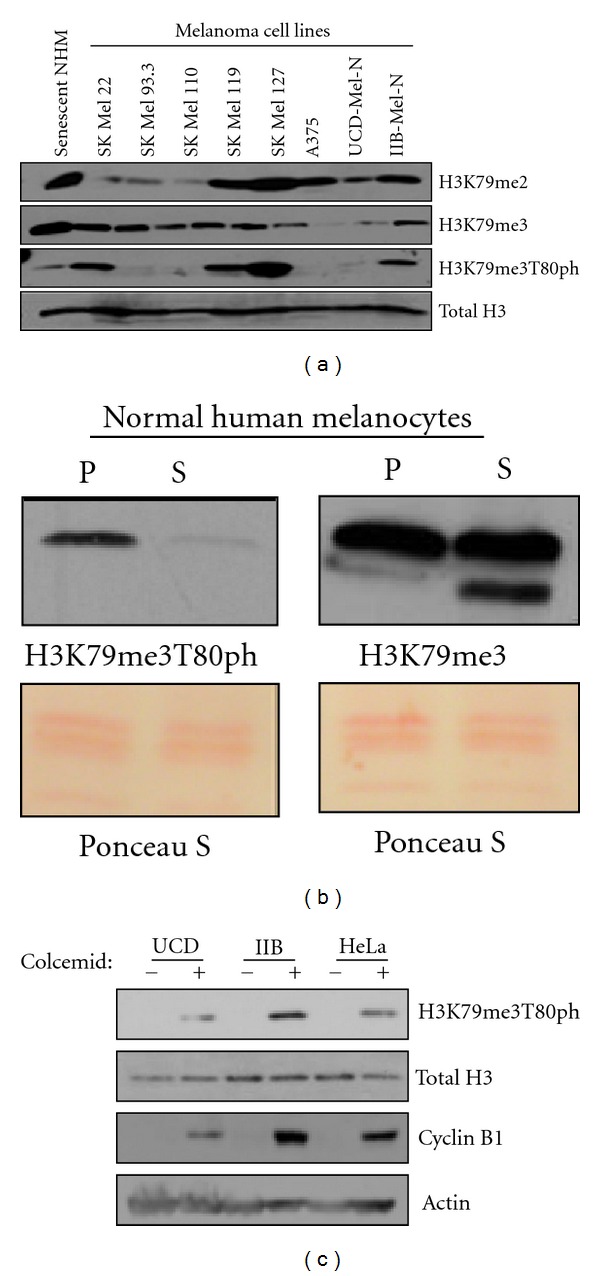
H3K79me3T80ph is cell cycle regulated. (a) H3K79me2, H3K79me3, and H3K79me3T80ph western blots of histones isolated from nondividing senescent human melanocytes and various human melanoma cell lines. (b) Western blot comparison of H3K79me3 and H3K79me3T80ph levels on histones from proliferating (P) and senescent (S) normal human melanocytes. (c) UCD-Mel-N, IIB-Mel-N, and HeLa cells were either asynchronous (−) or mitotically synchronized using colcemid (+). Histones were isolated to perform an H3K79me3T80ph western blot, and whole cell lysate was used to perform Cyclin B1 western blotting as a control for cell synchronization.

We next compared H3K79 methylation to H3K79me3T80ph patterns in various human melanoma cell lines and in nondividing senescent melanocytes. Western blot analysis of H3K79me2 and H3K79me3 identified cell-line specific patterns of H3K79 methylation (Figure 2(a)). This analysis also revealed that H3K79me3 and H3K79me3T80ph do not share a similar pattern of occurrence, indicating that these modifications are differentially regulated (Figure 2(a)). A similar pattern was observed in normal human melanocytes (NHMs), where H3K79me3 was found to be present in both proliferating and senescent NHMs, whereas H3K79me3T80ph was only detectable in proliferating NHMs (Figure 2(b)). Together this data suggest that H3K79me3T80ph is associated with cell proliferation; however, H3K79me3T80ph was undetectable in A375, SK Mel 93.3, SK Mel 110, and UCD-Mel-N melanoma cell lines paired with H3T80ph [15, 16]. 

### 3.3. H3K79me3T80ph is Cell Cycle Regulated

Given that H3K79me3T80ph is detectable only in proliferating cells, we sought to determine whether H3K79me3T80ph is cell-cycle regulated. HeLa and the human melanoma cell lines UCD-Mel-N and IIB-Mel-N were synchronized in mitosis with colcemid or vehicle treated [17, 18]. Synchronization was confirmed by Cyclin B1 western blot of whole cell extract (Figure 2(c)). H3K79me3T80ph levels were undetectable by western blot in asynchronous populations but increased significantly upon mitotic synchronization with colcemid, indicating that H3K79me3T80ph is indeed regulated in a cell-cycle-dependent manner (Figure 2(c)).

**Figure 3:**
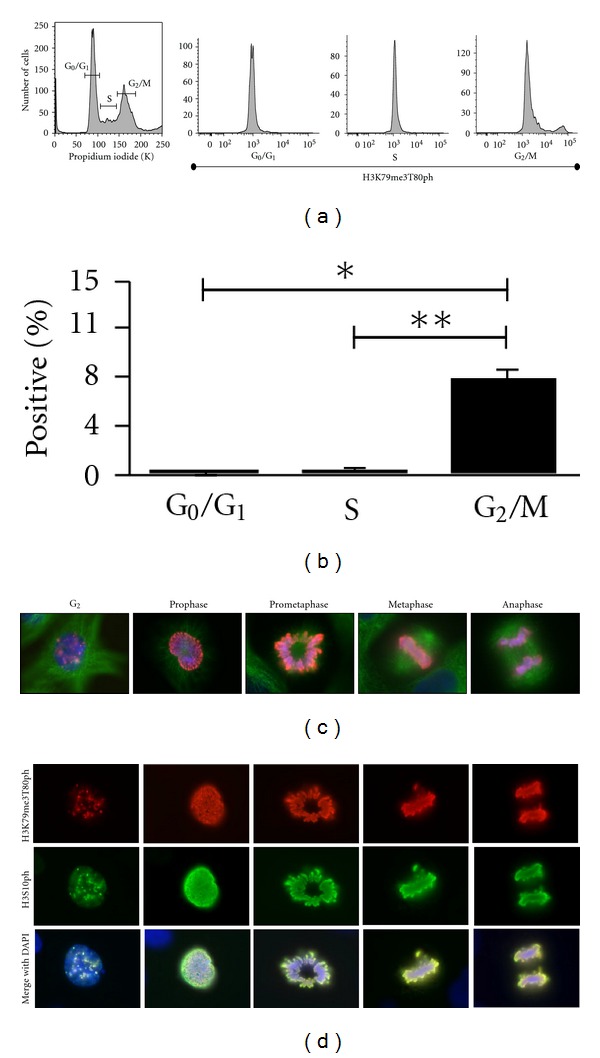
H3K79me3T80ph is mitosis-associated. (a) Representative flow cytometry experiment of untreated asynchronous HeLa cells stained with propidium iodide and H3K79me3T80ph (Area). Propidium iodide incorporation was used to obtain a cell cycle profile, and the cell cycle phases were then gated. An H3K79me3T80ph histogram was created for each phase of the cell cycle. (b) Percentage of H3K79me3T80ph positive cells in each cell cycle phase as determined by flow cytometry of asynchronous HeLa cells stained with propidium iodide and H3K79me3T80ph (Area). Bars represent mean values (± S.E.M.), *n* = 3; *p_G_0_/G_1_−G_2_/M_ = 0.0014, **p_S−G_2_/M_ = 0.0016. (C) Immunofluorescence of asynchronous UCD-Mel-N cells stained for H3K79me3T80ph (red), tubulin (green), and DNA (blue). (d) H3K79me3T80ph and H3S10ph co-immunofluorescence performed on asynchronous UCD-Mel-N cells; H3K79me3T80ph (red), H3S10ph (green), DNA (blue).

The dynamics of H3K79me3T80ph during the cell cycle were analyzed by flow cytometry. HeLa cells were indirectly stained for H3K79me3T80ph, and propidium iodide was used to determine DNA content. The cell-cycle phases were gated based on their DNA content, and the percentage of H3K79me3T80ph-positive cells was determined. Flow cytometry analysis revealed that G_0_/G_1_ and S phases have essentially undetectable levels of H3K79me3T80ph-positive cells (0.03% and 0.06%, resp.), whereas the number of H3K79me3T80ph-positive cells increased significantly in G_2_/M to 7.6% (*p*_G_0_/G_1_−G_2_/M_ = 0.0014, *p*_S−G_2_/M_ = 0.0016; Figures 3(a), and 3(b)). To verify that mitotic synchronization with colcemid increases the number of H3K79me3T80ph-positive cells, flow cytometry was repeated comparing the H3K79me3T80ph histogram in asynchronous and colcemid treated populations. The proportion of H3K79me3T80ph-positive cells in the population increased upon mitotic synchronization with colcemid, causing a positive shift in the H3K79me3T80ph histogram (data not shown).

### 3.4. H3K79me3T80ph is Primarily Associated with Mitotic Chromosomes

Having established that H3K79me3T80ph is G_2_/M associated, we next examined its localization *in vitro*. Immunofluorescence of H3K79me3T80ph in UCD-Mel-N cells demonstrates that H3K79me3T80ph is primarily associated with chromatin throughout mitosis (Figure 3(c)). Punctate H3K79me3T80ph staining was also observed in G_2_ cells with uncondensed DNA (Figure 3(c), first panel). 

H3S10ph is a well-studied mitotic histone modification that appears to share the similar dynamic as H3K79me3T80ph persisting on chromosomes until anaphase [7]. To gain insight into the regulation of H3K79me3T80ph, we compared the spatial-temporal regulation of H3K79me3T80ph to that of H3S10ph. Coimmunofluorescence in UCD-Mel-N cells demonstrated that H3K79me3T80ph and H3S10ph colocalize completely throughout mitosis (Figure 3(d)). This colocalization also occurs prior to chromosome condensation, suggesting that, like H3S10ph, H3K79me3T80ph appears in late G_2_ [7].

### 3.5. Aurora B is Necessary for Catalysis of H3K79me3T80ph

**Figure 4:**
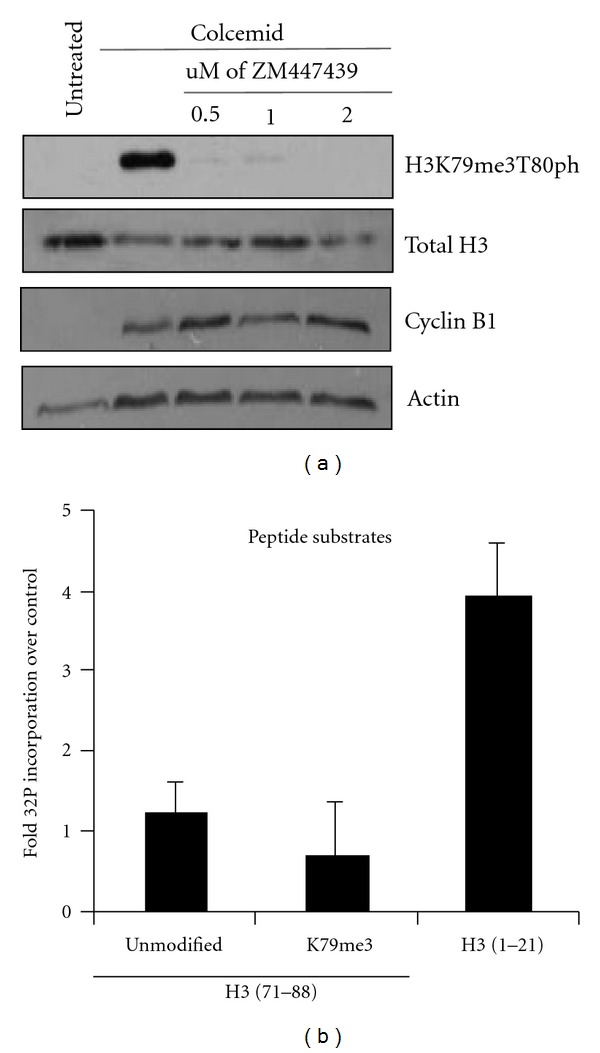
Aurora B is required for H3T80 phosphorylation. (a) HeLa cells were subjected to no treatment, colcemid arrest, and colcemid arrest in conjunction with ZM447439 treatment. Half of the cells were used to isolate histones and to perform an H3K79me3T80ph western blot, and half of the cells were used to make whole-cell lysate and perform a Cyclin B1 western to control for synchronization. (b) *In vitro* kinase assay using recombinant Aurora B/INCENP in the presence of ^32^P-gamma-ATP and biotinylated H3 (1–21); H3 (71–88); H3 (71–88), K79me3 peptides. Bars represent fold mean ^32^P scintillation counts over cold phosphorylated peptide (±S.E.M.), *n* = 3.

Aurora B is a well-described mitotic kinase that catalyzes N-terminal H3 mitotic phosphorylation at H3S10ph and H3S28ph [7]. Having seen that H3K79me3T80ph appears to share the same pattern of regulation as H3S10ph, we examined if Aurora B is involved in H3K79me3T80ph regulation by culturing HeLa cells in the presence of colcemid and ZM447439, a potent Aurora B inhibitor [19]. Colcemid treatment alone resulted in a robust H3K79me3T80ph signal; however, this detection was loss in the presence of ZM447439 (Figure 4(a)). It has been reported previously that although ZM447439 inhibits Aurora B, treated cells can still exit mitosis with normal dynamics [19]. Cyclin B1 western blot of whole cell extract from cells treated with colcemid and ZM447439 treatment verifies that the cells were arrested in mitosis; therefore, loss of H3K79me3T80ph is not due to mitotic exit (Figure 4(a)).

To directly address whether Aurora B can phosphorylate H3T80, an *in vitro* kinase assay was performed using recombinant human Aurora B and INCENP, a protein important for stimulating Aurora B kinase activity [20]. Aurora B/INCENP was incubated with biotinylated H3 peptides (H3 (71–88), H3 (71–88) K79me3, and H3 (1–21)) in the presence of ^32^P-ATP. The level of  ^32^P incorporation was determined by scintillation counting, which was compared to peptides incubated with cold phosphate (H3 (71–88) T80ph, H3 (71–88) K79me3T80ph, and H3 (1–21) S10ph. Recombinant Aurora B/INCENP robustly phosphorylated the H3 (1–21) peptide, presumably on S10, but was unable to phosphorylate the H3 (71–88) peptide to the same extent, whether the peptide was unmodified or trimethylated (Figure 4(b)). Together these results indicate that Aurora B/INCENP is necessary for T80 phosphorylation *in vivo*, but not sufficient for H3T80 phosphorylation in an *in vitro* peptide kinase assay. Aurora B is a member of several protein complexes and may require additional proteins to target its activity toward H3T80 *in vivo*. However, the H3 (71–88) peptide used here derives from the core domain of H3 and, *in vitro*, may not possess the structure or sufficient residues for proper T80 phosphorylation, unlike the H3 (1–21) peptide which likely exists in an unordered state *in vitro* as the H3 tail does *in vivo* making it suitable for Aurora B activity.

Previous reports have demonstrated that phosphorylation of H3T3 by Haspin greatly increases Aurora B activity toward H3S10 and H3S28. Similar to H3T80, H3T3 lies adjacent to a well-described methylation mark at H3K4; therefore, we wanted to determine if Haspin was capable of phosphorylating H3T80. *In vitro* kinase assays using recombinant Haspin and biotinylated H3 (1–21) and H3 (71–88) were performed in the presence of ^32^P-ATP. Consistent with published reports, Haspin was capable of phosphorylating the H3 (1–21) peptide. However, Haspin was unable to phosphorylate the H3 (71–88) peptide, demonstrating that Haspin, like Aurora B, is not sufficient for the phosphorylation of H3T80 (data not shown).

### 3.6. H3K79me3T80ph is an Indicator of Tumor Proliferation and Mitosis *In Vivo*

**Figure 5:**
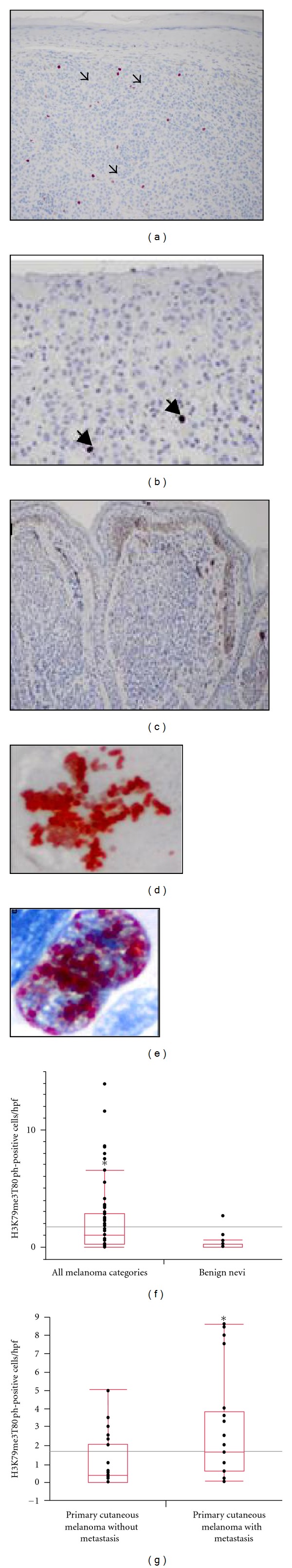
Immunohistochemistry (IHC) H3K79me3T80ph detects a subset of primary cutaneous melanomas with metastatic potential. Positive H3K79me3T80ph cells (arrows) in: (a) metastatic melanoma of the skin (magnification: ×100) and (b) primary cutaneous invasive melanoma (magnification: ×200) (c) Melanocytic nevi with absence of H3K79me3T80ph labeling (magnification: ×100). (d) and (e) Representative H3K79me3T80ph staining of melanoma tissue microarray (d): mitotically active cells, (e): melanoma cells with speckled nuclear staining pattern (Magnification: ×400). (f) and (g) Box graph analysis of the number of H3K79me3T80ph positive cells per high powered field (h.p.f.) within the melanocytic neoplasms contained in the tissue microarray. (f) Melanoma from all categories demonstrates greater number H3K79me3T80ph positive cells compared to benign nevi (*P* = 0.0019). (g) Significant labelling of H3K79me3T80ph positive cells in primary cutaneous melanomas with known metastasis compared to primary melanomas without metastasis (*P* = 0.0044).

We next sought to determine if H3K79me3T80ph staining was capable of detecting proliferation differences in human melanoma samples using a tissue microarray. The array included 87 lesions consisting of benign melanocytic nevi, primary cutaneous melanomas with and without known metastasis, and metastatic melanomas. The array was stained for H3K79me3T80ph, and positive cells per high-power field were counted. H3K79me3T80ph was primarily observed in a speckled nuclear pattern and on chromosomes of mitotically active cells within the melanoma sections but was not detectable in melanocytic nevi (Figures 5(a), 5(b), and 5(c)). Further analysis demonstrated that the average number of H3K79me3T80ph-positive cells per high-powered field was significantly higher in all subcategories of melanomas when compared to benign melanocytic nevi (Figure 5(f), *P* = 0.0019). Notably the number of H3K79me3T80ph-positive cells in primary cutaneous melanoma with metastasis was significantly higher when compared to primary melanomas without metastasis (Figure 5(g), *P* = 0.0044). The number of H3K79me3T80phpositive cells was similar between primary melanoma with metastasis and in metastatic melanoma. Analysis for H3K79me3T80ph in the metastatic melanoma category included cases that clinically and histologically represented metastasis and not local recurrences. However, recurrent tumors or tumors which reappeared after surgical removal may in some clinical situations represent a metastasis. Since there was no difference in the labeling of H3K79me3T80ph in primary tumors with known metastasis from metastatic melanoma, one could speculate that primary tumors with known metastasis and “recurrent tumors” will likely demonstrate similar amount of H3K79me3T80ph-positive cells; however, further studies are needed for confirmation. There was no significant difference in the intensity of H3K79me3T80ph labelling between the melanoma categories.

## 4. Conclusion

The interplay between histone modifications is the foundation of the histone code hypothesis [21]. Through the use of cell synchronization experiments, flow cytometry, and immunofluorescence studies, we have found that H3K79me3T80ph is a novel histone dual modification in the core of histone H3. H3K79me3T80ph becomes visible prior to the onset of obvious chromosome condensation. Therefore, it is likely that the dual modification appears in G_2_. This is further supported by our immunofluorescence studies showing that H3K79me3T80p shares the same temporal regulation as H3S10ph, which is known to appear in late G_2_ [7]. However, our investigation into the regulation of H3K79me3T80ph revealed that, unlike the N terminus of histone H3, Aurora B is not sufficient to catalyze H3T80ph *in vitro* although Aurora kinase activity is required for the catalysis of H3K79me3T80ph. Considering that there are many mitotic kinases, H3T80 phosphorylation may be catalyzed by an unidentified kinase that may require Aurora B for its own activation, which would explain why the inhibition of Aurora kinase activity via ZM447439 treatment results in loss of H3K79me3T80ph. However, it is also reasonable to speculate that Aurora B *in vivo* can phosphorylate H3T80 but requires additional proteins to target Aurora B kinase activity toward H3T80. A similar instance has been observed for H3S10ph and survivin. Survivin is a member of the chromosome passenger complex with Aurora B and INCEP, and upon phosphorylation of H3T3 survivin directs Aurora B activity toward H3S10ph [22, 23]. However, even in the absence of survivin Aurora B shows activity toward H3S10, which is not the case for H3T80. Furthermore, the sequence surrounding H3K79/T80 and F78-K79-T80, does not match the Aurora B consensus sequence (R/K-X-S/T), meaning that Aurora B is not predicted to be a T80 kinase but likely lies upstream of the H3T80 kinase [24]. 

In addition, H3K79me3T80ph appears to identify a subset of primary cutaneous melanomas with metastatic potential. Melanoma is the deadliest of all skin cancers, and proper diagnosis and risk stratification are important for treatment and prognosis. Mitotic rate in primary cutaneous melanoma has been determined to be the second most powerful predictor of survival, immediately after tumor (Breslow) thickness [25]. Ancillary IHC studies with H3S10ph (PHH3) have been shown to be an additional clinical tool to evaluate for mitoses in melanoma [26]. The value of histone H3 N-terminal modifications as a prognostic marker of invasive melanoma in a clinical setting is emerging; however, the utility of specific proliferative markers as prognostic indicator appears context dependent. For instance, a study by Ladstein et al. examined H3S10ph (PHH3) in nodular invasive melanomas and found that although H3S10ph is a mitotic marker, it did not demonstrate prognostic value for nodular melanomas [27]. We have detected H3K79me3T80ph in primary cutaneous melanomas, and it appears to identify a subset of primary melanomas with metastatic potential. Additional studies on separate cohorts may be needed to determine possible clinical applications, but our results reveal a possible use of H3K79me3T80ph as a biomarker for the identification of primary cutaneous melanoma with more aggressive clinical behaviour.
